# Neural Substrate of Cold-Seeking Behavior in Endotoxin Shock

**DOI:** 10.1371/journal.pone.0000001

**Published:** 2006-12-20

**Authors:** Maria C Almeida, Alexandre A Steiner, Luiz G S Branco, Andrej A Romanovsky

**Affiliations:** 1 Systemic Inflammation Laboratory, Trauma Research, St. Joseph's Hospital and Medical Center Phoenix, Arizona, United States of America; 2 Medical School of Ribeirão Preto, University of São Paulo Ribeirão Preto, São Paulo, Brazil; 3 Dental School of Ribeirão Preto, University of São Paulo Ribeirão Preto, São Paulo, Brazil; Duke University Medical Center, United States of America

## Abstract

Systemic inflammation is a leading cause of hospital death. Mild systemic inflammation is accompanied by warmth-seeking behavior (and fever), whereas severe inflammation is associated with cold-seeking behavior (and hypothermia). Both behaviors are adaptive. Which brain structures mediate which behavior is unknown. The involvement of hypothalamic structures, namely, the preoptic area (POA), paraventricular nucleus (PVH), or dorsomedial nucleus (DMH), in thermoregulatory behaviors associated with endotoxin (lipopolysaccharide [LPS])-induced systemic inflammation was studied in rats. The rats were allowed to select their thermal environment by freely moving in a thermogradient apparatus. A low intravenous dose of *Escherichia coli* LPS (10 µg/kg) caused warmth-seeking behavior, whereas a high, shock-inducing dose (5,000 µg/kg) caused cold-seeking behavior. Bilateral electrocoagulation of the PVH or DMH, but not of the POA, prevented this cold-seeking response. Lesioning the DMH with ibotenic acid, an excitotoxin that destroys neuronal bodies but spares fibers of passage, also prevented LPS-induced cold-seeking behavior; lesioning the PVH with ibotenate did not affect it. Lesion of no structure affected cold-seeking behavior induced by heat exposure or by pharmacological stimulation of the transient receptor potential (TRP) vanilloid-1 channel (“warmth receptor”). Nor did any lesion affect warmth-seeking behavior induced by a low dose of LPS, cold exposure, or pharmacological stimulation of the TRP melastatin-8 (“cold receptor”). We conclude that LPS-induced cold-seeking response is mediated by neuronal bodies located in the DMH and neural fibers passing through the PVH. These are the first two landmarks on the map of the circuitry of cold-seeking behavior associated with endotoxin shock.

## Introduction

Deep body temperature (T_b_) is regulated by both autonomic and behavioral means. Autonomic thermoregulation is limited in its ability to compensate for thermal loads and exacts a high price. Indeed, the most effective mechanisms of heat loss involve evaporation of water from the surface of the skin or respiratory pathways (e.g., by sweating, salivation, or polypnea), which strains the body's precious water resources, whereas heat production (nonshivering or shivering thermogenesis) depletes the body's energy stores. In contrast to autonomic thermoregulation, behavior places no demands on the body's water or energy resources; furthermore, behavioral thermoregulation can compensate for much greater thermal loads. By behavioral means, humans can survive at ambient temperatures (T_a_s) ranging from −110°C (the surface of the moon) to 2,000°C (the air around a space shuttle as it reenters the atmosphere) while maintaining T_b_ within a few tenths of a degree Celsius [1]. Behavioral thermoregulatory responses vary from primitive (e.g., locomotion to a preferred T_a_ in a T_a_ gradient) to complex (e.g., maintaining T_a_ inside a space shuttle). Evidence (mostly from stimulation experiments) suggests that different thermoregulatory behaviors in the rat (e.g., relaxed postural extension, thermoregulatory grooming, and locomotion) use distinct neural circuitries [2]. However, the neuroanatomic substrate of no thermoregulatory behavior has been studied extensively, and almost nothing is known about the neuroanatomy of behavioral thermoregulation [3].

The present study was undertaken to evaluate whether hypothalamic structures are involved in selection of preferred T_a_ under various conditions in rats. We were especially interested in the thermoregulatory behavior associated with systemic inflammation. In the laboratory, systemic inflammation is often studied by administering bacterial lipopolysaccharide (LPS, endotoxin) to rats. Studies using this model have suggested that low doses of LPS (mild inflammation) cause fever and warmth-seeking behavior [4], [5], whereas high doses (severe inflammation) cause hypothermia and cold-seeking behavior [5], [6]. Warmth-seeking behavior in mild systemic inflammation is likely to contribute to the development of fever, an increase in T_b_ that exerts antimicrobial and immunostimulating actions [7]. Cold-seeking behavior and hypothermia occurring in severe systemic inflammation are also beneficial [8]. They are aimed at energy conservation and are associated with analgesia, sleep, and locomotor depression, i.e., the energy-saving symptoms proposed or demonstrated to be beneficial during infection and severe inflammation [9]–[11]. With the exception of one study in toads [12], the neuronal circuitry of LPS-induced warmth-seeking behavior has not been investigated, and nothing is known about brain mediation of LPS-induced cold-seeking behavior.

## Results and Discussion

### Hypothalamic structures studied

**Figure 1:**
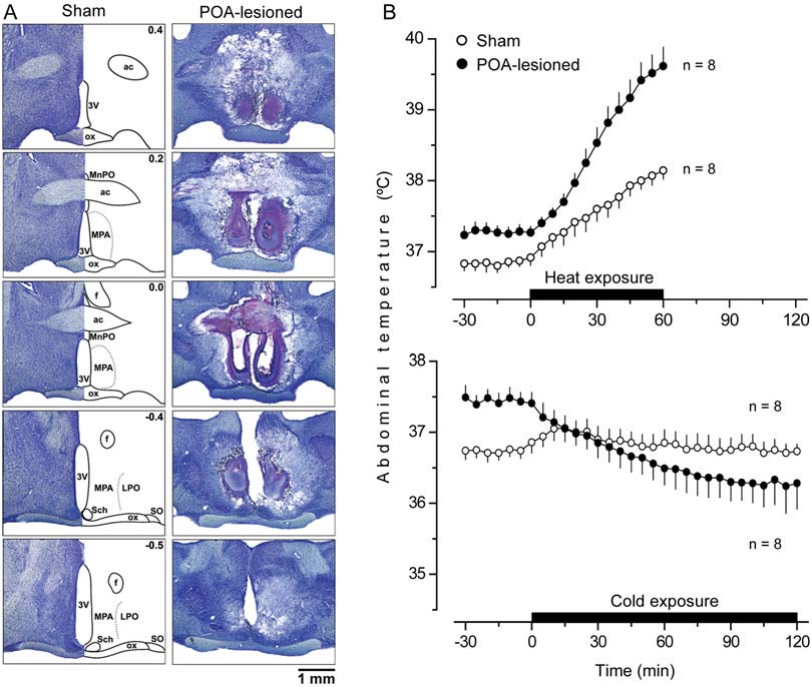
Electrolytic ablation of the POA: histological verification and effects on autonomic thermoregulation. (A) Bright-field photomicrographs of serial coronal brain sections (50 µm, cresyl violet staining) are shown for a sham-lesioned rat and a POA-lesioned rat. Here and in Figures 2, [3], [7] and [8], the number in the right upper corner of the schematic of each section of the sham-lesioned brain indicates the distance (in mm) between the section's plane and bregma. ac, anterior commissure; f, fornix; LPO, lateral preoptic area; MnPO, median preoptic nucleus; MPA, medial preoptic area; ox, optic chiasm; Sch, suprachiasmatic nucleus; SO, supraoptic nucleus; 3V, third ventricle. (B) The ability of sham-lesioned and POA-lesioned rats to defend their T_b_ (abdominal) during moderate heat exposure (28°C, 1 h) or mild cold exposure (17°C, 2 h). The rats could not move to a different T_a_; therefore, they were forced to regulate their T_b_ mostly by autonomic mechanisms.

Three structures were selected for this study: the preoptic area (POA), paraventricular hypothalamic nucleus (PVH), and dorsomedial hypothalamic nucleus (DMH). The POA, previously known as the “thermoregulatory center,” contains warm-sensitive neurons that control all autonomic thermoeffectors [3] and that are thought to be the first neurons in the efferent fever pathway [13]–[16]. Animals with POA lesions cannot defend their T_b_ autonomically against either cold or heat [17]–[21]. In the present study, we placed large bilateral electrolytic lesions in the POA of rats (Fig. 1A). Confirming findings by others [17]–[25], the POA-lesioned rats had a somewhat elevated T_b_ and were incapable of defending their T_b_ autonomically against either moderate heat exposure (P = 0.003) or mild cold exposure (P<0.001) (Fig. 1B). In fact, heat-induced hyperthermia in POA-lesioned animals was so severe that the time of heat exposure in these experiments had to be reduced to 1 h (instead of the 2 h planned) to avoid heat stroke.

**Figure 2:**
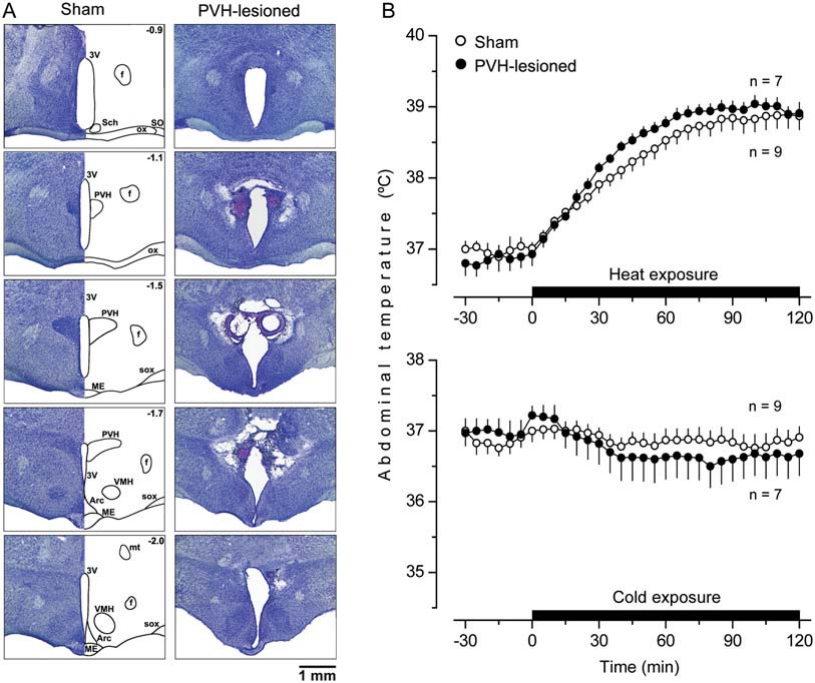
Electrolytic ablation of the PVH: histological verification and effects on autonomic thermoregulation. (A) Serial coronal brain sections are shown for a sham-lesioned rat and a PVH-lesioned rat. Arc, arcuate hypothalamic nucleus; ME, median eminence; mt, mammillothalamic tract; sox, supraoptic decussation; VMH, ventromedial hypothalamic nucleus. Other abbreviations used are the same as in Figure 1. (B) The ability of sham-lesioned and PVH-lesioned rats to defend their T_b_ by autonomic mechanisms during moderate heat exposure (28°C, 2 h) or mild cold exposure (17°C, 2 h).

The second structure studied, the PVH, is unique from a thermoregulatory perspective: it has been implicated in selectively mediating thermoregulatory responses to inflammatory stimuli. Indeed, electrolytic and chemical lesions of the PVH neither alter the circadian rhythm of T_b_ [26] nor affect T_b_ responses to cold or heat [27], [28]. However, they do attenuate LPS fever, at least when the animals cannot use behavioral means to regulate their T_b_ and must rely on autonomic means to mount the febrile response [26]–[28]. We placed large bilateral electrolytic lesions in the PVH of rats (Fig. 2A). As expected, such lesions did not affect their ability to defend T_b_ autonomically against either heat or cold (Fig. 2B).

**Figure 3:**
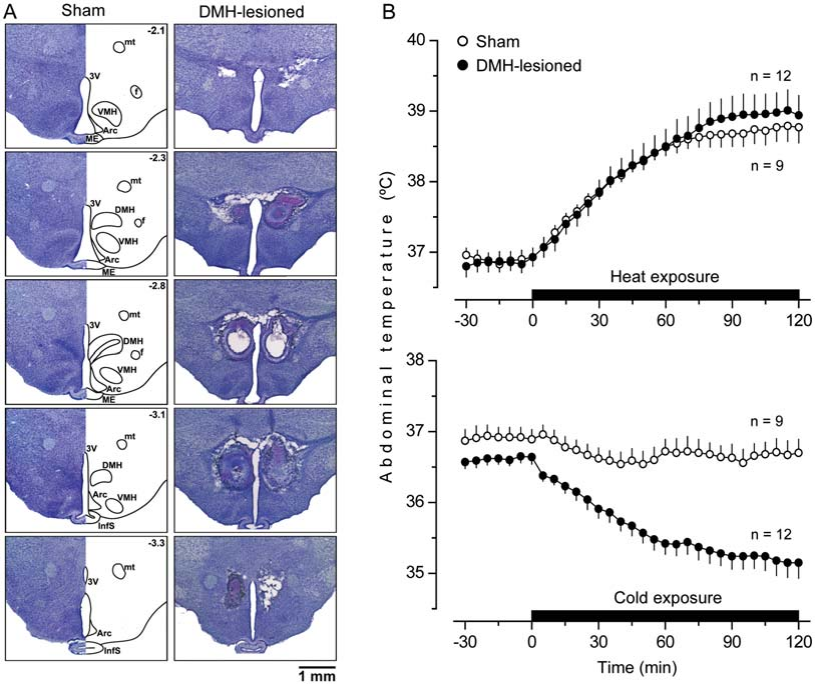
Electrolytic ablation of the DMH: histological verification and effects on autonomic thermoregulation. (A) Serial coronal brain sections are shown for a sham-lesioned rat and a DMH-lesioned rat. InfS, infundibular stem. Other abbreviations used are the same as in Figures 1 and [2]. (B) The ability of sham-lesioned and DMH-lesioned rats to defend their T_b_ by autonomic mechanisms during moderate heat exposure (28°C, 2 h) or mild cold exposure (17°C, 2 h).

The third structure studied, the DMH, is involved in the control of the most important heat production thermoeffector in the rat: brown adipose tissue [3], [29], [30]. Blocking the activation of DMH neurons pharmacologically interrupts the stimulation of brown fat thermogenesis [31], [32]. A recent study of Fos protein expression in the brain [33] suggested that the DMH is also involved in the control of operant heat-avoidance behavior (moving to a reward area to trigger a breeze of cold air). When we placed large bilateral electrolytic lesions in the DMH (Fig. 3A), the lesions did not affect the autonomic defense of T_b_ against heat. However, they strongly compromised autonomic cold-defense: the same cold exposure that failed to alter T_b_ of the sham-lesioned rats caused marked hypothermia (P<0.001) in DMH-lesioned rats (Fig. 3B).

### Electrolytic lesions: effects on thermoregulatory behavior

**Figure 4:**
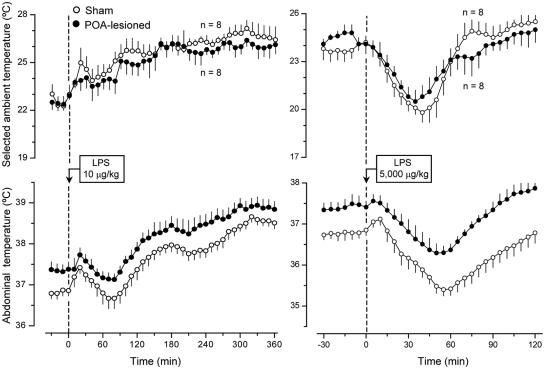
The effects of LPS (doses indicated) on the selected T_a_ (top panels) and T_b_ (bottom panels) of sham-lesioned and POA-lesioned rats.

After we verified that lesions of different hypothalamic structures produced the expected effects on the autonomic regulation of T_b_, we studied the effects of these lesions on thermoregulatory behavior in a thermogradient apparatus. Systemic inflammation was induced by intravenous (i.v.) injection of bacterial LPS either at a low, fever-inducing dose (10 µg/kg) or at a high, shock- and hypothermia-inducing dose (5,000 µg/kg). These two doses cause, respectively, slowly occurring, long-lasting warmth-seeking behavior and rapidly occurring, marked cold-seeking behavior [5]. Although all autonomic responses were severely compromised in the POA-lesioned rats (Fig. 1B), neither LPS-induced warmth-seeking behavior nor LPS-induced cold-seeking behavior was affected in these animals (Fig. 4). Furthermore, when allowed to regulate their T_b_ behaviorally in the thermogradient apparatus, the POA-lesioned rats responded to a low dose of LPS with normal fever, and to a high dose of LPS with normal hypothermia (Fig. 4). These findings are somewhat unexpected. They seem to contradict the current view that neuronal groups within the preoptic anterior hypothalamus are crucial for generating the febrile response [15], [34], [35]. They are also contrary to the finding that lesioning the POA attenuates LPS-induced warmth-seeking behavior in toads [12]. However, squirrel monkeys [36] and rabbits [25] have been shown to develop normal febrile responses to LPS and prostaglandin E_1_ when the POA is ablated electrolytically. The two latter studies and the present one indicate that an intact POA is not required for LPS fever, thus suggesting that POA neurons are not the only targets for febrigenic mediators. That the febrile response can occur when the POA is ablated bilaterally suggests that the current understanding of the neural basis of fever has to be revised. The present study also shows that LPS hypothermia can occur when the POA is coagulated.

We also looked at thermoregulatory locomotion induced by non-inflammatory stimuli: thermal and pharmacological. Thermal stimuli included mild cold exposure and moderate heat exposure (see Methods for details). Pharmacological stimulation was achieved by systemic administration of agonists of the so-called thermo-TRP (transient receptor potential) channels, a class of heat-activated molecules recently implicated in thermosensation [37]–[40]. At least some members of this class have been shown to be involved in the selection of preferred T_a_ in both vertebrates [5], [41], [42] and invertebrates [43]. In the present study, we used menthol (50 µg/kg, i.v.), an agonist of the TRP melastatin-8 (TRPM8) channel (“cold receptor”), to induce warmth-seeking behavior and resiniferatoxin (RTX, 0.5 µg/kg, i.v.), an agonist of the TRP vanilloid-1 (TRPV1) channel (“warmth receptor”), to induce cold-seeking behavior. All four stimuli (two thermal and two pharmacological) were also used in our recent study, which describes thermoregulatory behaviors of normal rats to these stimuli [5]. In the present study, none of the four behavioral responses studied was affected in the POA-lesioned rats (data not shown). That the POA lacks an indispensable role in warmth- and cold-seeking behaviors induced by thermal and pharmacological stimuli is consistent with the literature. Indeed, the only mammalian thermoregulatory behavior in which involvement of the POA has been firmly established is a relaxed postural extension in response to heat exposure; such postural extension does not occur in POA-lesioned animals [44]. Other thermoregulatory behaviors, such as moving to a “reward” zone or pressing a lever to trigger warming or cooling of the system, remain intact in POA-lesioned animals [20], [21], [45], [46]. That ablation of the POA results in the loss of autonomic responses but does not affect thermoregulatory locomotion suggests that POA thermosensors are more important for autonomic thermoregulation than for cold- and warmth-seeking behaviors. Because thermoregulatory locomotion is aimed at escaping the forthcoming thermal insult, it occurs before the body core warms up or cools down; therefore, it is triggered by peripheral temperatures. In contrast, autonomic cold-defense responses (energetically expensive) and heat-defense responses (water-consuming) are often recruited only when T_b_ starts changing because behavioral mechanisms were ineffective or could not have been used (e.g., due to competing behavioral demands) [1].

**Figure 5:**
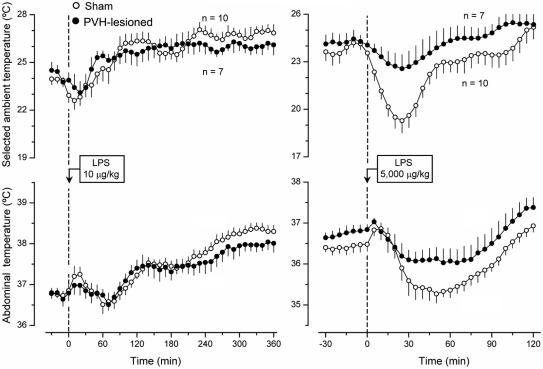
The effects of LPS (doses indicated) on the selected T_a_ (top panels) and T_b_ (bottom panels) of sham-lesioned and PVH-lesioned rats.

Like the electrolytic lesions of the POA, lesions of the PVH affected neither warmth-seeking behavior nor fever induced by a low dose of LPS. Unlike the POA lesions, lesions of the PVH strongly attenuated both cold-seeking behavior (P = 0.035) and hypothermia (P = 0.047) caused by a high, shock-inducing dose of LPS (Fig. 5). The observation that lesions of the PVH had no effect on the LPS-induced warmth-seeking behavior and fever is somewhat surprising, because electrolytic and excitotoxic lesions of this structure have attenuated the febrile response to LPS in several studies [26]–[28]. However, the animals used in these previous studies were not allowed to select their preferred T_a_. Furthermore, the previous studies were most likely conducted under subthermoneutral conditions (for detailed discussion of thermoneutrality, see Ref. [47]). Under such conditions, fever is caused primarily by activation of brown fat thermogenesis [48], [49]. In contrast, PVH-lesioned rats were allowed to select their preferred T_a_ and used behavioral thermoregulation (moved to a warmer environment) while responding to LPS administration in the present study. Not only does a supraneutral environment warms the body of a rat exposed to it, but it also allows the animal to mount the fever response by using skin vasoconstriction instead of the energetically expensive thermogenesis [48], [49]. Hence, the PVH is likely involved in the circuitry of fever when the response is mounted primarily by activation of brown fat thermogenesis. When fever occurs due to warmth-seeking behavior, skin vasoconstriction, or both, the PVH loses its important role in the response. Such a scenario is consistent with the fact that the PVH controls primarily thermogenesis and not skin vasomotion [50]–[52] or thermopreferendum (present study). The effect of PVH ablation on LPS-induced cold-seeking behavior was highly selective: the same electrolytic lesions that strongly attenuated this behavior affected neither cold-seeking behavior caused by moderate heat exposure or RTX nor warmth-seeking behavior caused by mild cold exposure or menthol (data not shown).

**Figure 6:**
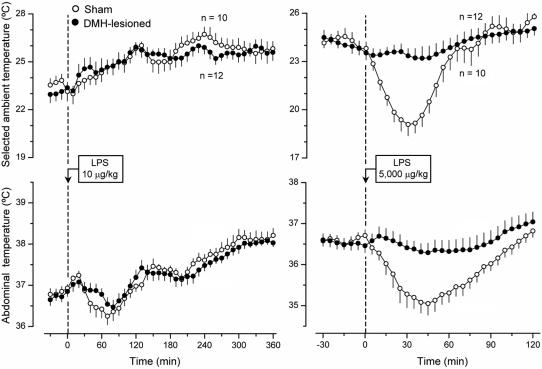
The effects of LPS (doses indicated) on the selected T_a_ (top panels) and T_b_ (bottom panels) of sham-lesioned and DMH-lesioned rats.

The effects of lesioning the DMH were remarkably similar to those of lesioning the PVH. Electrocoagulation of the DMH blocked both cold-seeking behavior (P = 0.04) and hypothermia (P = 0.032) caused by a high, shock-inducing dose of LPS, but it affected neither warmth-seeking behavior nor fever caused by a low dose of LPS (Fig. 6). Neither did electrocoagulation of DMH affect cold-seeking behaviors caused by heat exposure and menthol nor warmth-seeking behaviors caused by cold exposure and RTX (data not shown). The found attenuation of LPS-induced hypothermia in PVH- and DMH-lesioned rats is a first report of a brain structure being crucial for the development of hypothermia in systemic inflammation. These findings provide an additional, perhaps decisive, argument in a dispute as to whether LPS-induced hypothermia is a passive consequence of peripheral vasodilation and uncontrolled heat loss or, alternatively, whether it is a brain-mediated response. To determine whether the effects of electrolytic lesioning of the PVH and DMH on cold-seeking behavior and hypothermia in LPS shock were from the destruction of neuronal bodies in these areas or, alternatively, from the interruption of fibers of passage, we performed chemical lesions with ibotenic acid, an excitotoxin known to destroy neuronal bodies but to spare passing axons [53].

### Excitotoxic lesions of PVH and DMH: effects on thermoregulation

**Figure 7:**
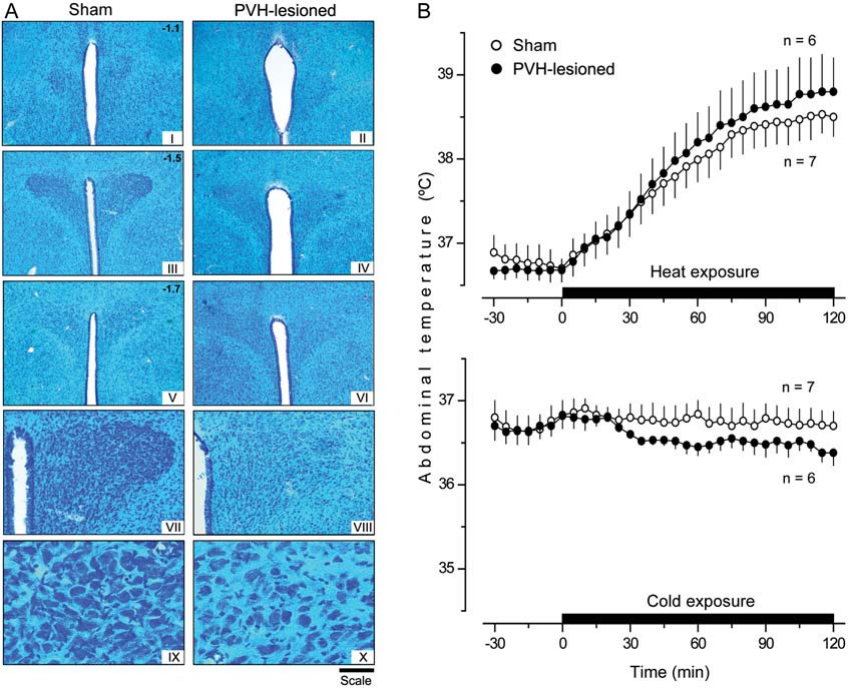
Excitotoxic ablation of the PVH: histological verification and effects on autonomic thermoregulation. (A) Bright-field photomicrographs of serial coronal brain sections (50 µm, Klüver-Barrera staining) of a sham-lesioned rat and a representative rat with bilateral ibotenic acid lesions of the PVH. The magnification of photomicrographs and the scale vary in different panels. Panels I-VI: ×40 (magnification) and 500 µm (scale bar). Panels VII and VIII: ×100 (magnification) and 200 µm (scale bar). Panels IX and X: ×400 (magnification) and 50 µm (scale bar). (B) The ability of sham-lesioned rats and rats with bilateral ibotenic acid lesions of the PVH to defend their T_b_ by autonomic mechanisms during moderate heat exposure or mild cold exposure.

**Figure 8:**
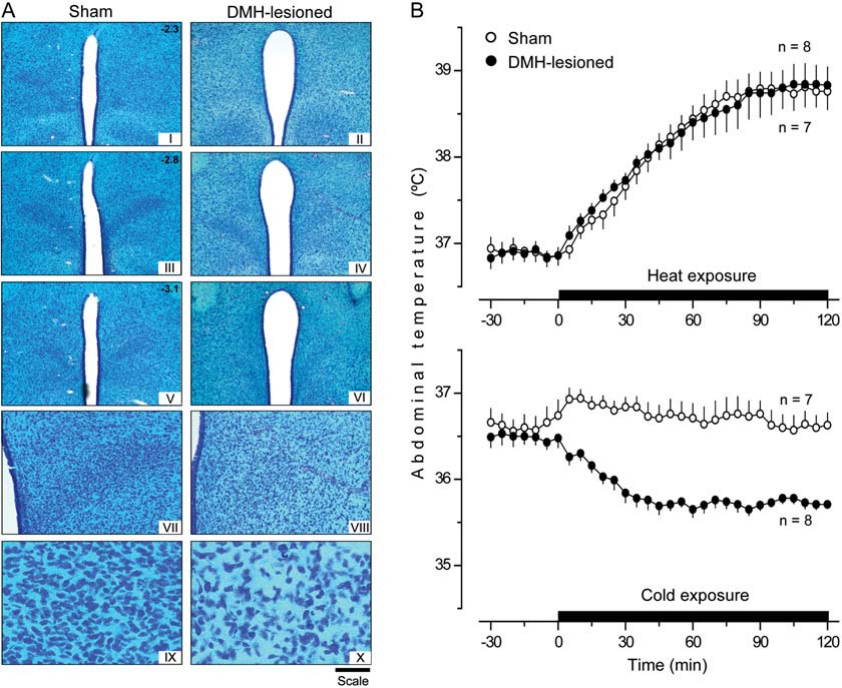
Excitotoxic ablation of the DMH: histological verification and effects on autonomic thermoregulation. (A) Bright-field photomicrographs of serial coronal brain sections (50 µm, Klüver-Barrera staining) of a sham-lesioned rat and a representative rat with bilateral ibotenic acid lesions of the DMH. The magnification and scale of each panel are the same as those of the corresponding panel in Figure 7. (B) The ability of sham-lesioned rats and rats with bilateral ibotenic acid lesions of the DMH to defend their T_b_ by autonomic mechanisms during moderate heat exposure or mild cold exposure.

**Figure 9:**
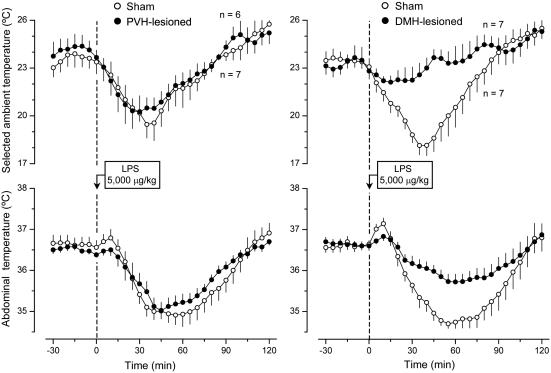
The effects of LPS (5,000 µg/kg, i.v.) on the selected T_a_ and T_b_ of sham-lesioned rats and rats with bilateral ibotenic acid lesions of either PVH or DMH.

Similarly to how electrocoagulation of the PVH (Fig. 2A) did not affect autonomic thermoregulation (Fig. 2B), bilateral lesioning of this structure by ibotenic acid (Fig. 7A) had no effect on autonomic heat-defense or cold-defense mechanisms (Fig. 7B). Similarly to how electrolytic lesions of the DMH (Fig. 3A) did not affect autonomic heat-defense mechanisms but impaired autonomic cold-defense mechanisms (Fig. 3B), bilateral ibotenic acid lesioning of the DMH (Fig. 8A) had no effect on the autonomic defense of T_b_ during heat exposure but made the rats incapable of defending their T_b_ autonomically against mild cold exposure (P<0.001; Fig. 8B). The effects of excitotoxic lesions of these two structures on responses to the high dose of LPS were different (Fig. 9). Lesioning the PVH affected neither LPS-induced cold-seeking behavior nor LPS-induced hypothermia. In contrast, lesioning the DMH by ibotenic acid prevented LPS-induced cold-seeking behavior (P = 0.014) and attenuated LPS hypothermia (P = 0.028). These results indicate that the DMH contains the bodies of neurons involved in the mechanisms of cold-seeking behavior and hypothermia caused by a high dose of LPS, and that the PVH contains neuronal fibers (but not cell bodies) involved in these two responses.

How the DMH neurons and PVH fibers fit into the circuitry of LPS-induced cold-seeking behavior is a subject for speculation. The DMH projects to the periaqueductal gray matter (PAG) [54], [55]. The PAG is a center for efferent integration of autonomic and behavioral responses, and different cellular groups within the PAG are involved in motor behaviors caused by various stimuli [56], [57]. It is, therefore, tempting to hypothesize that DMH lesions block LPS-induced cold-seeking behavior by interrupting the DMH projection to the PAG. However, this projection does not go through the PVH. Hence, the DMH-PAG hypothesis cannot explain the effect of PVH lesions. Furthermore, cold-seeking responses triggered by different stimuli represent the same behavioral act (moving to a lower T_a_) and are, therefore, likely to share the same efferent (motor) pathway. Yet, our data show that the involvement of DMH neurons and of PVH fibers of passage in cold-seeking behavior is specific to stimulation with a high dose of LPS. Indeed, the integrity of neither structure is needed to mount cold- or warmth-seeking behavior induced by any of the other five stimuli tested in the present study. Therefore, involvement of the DMH-PAG projection seems improbable.

Because rats with DMH or PVH lesions respond normally to thermal stimulation (cold or warmth) and to pharmacological stimulation of either a cold (TRPM8) or a warmth (TRPV1) receptor, both DMH and PVH are likely uninvolved in the thermoafferent pathway. This pathway (also known as the interoceptive afferent system) includes, most notably, the parabrachial nucleus and insular cortex [58], [59]. Although a direct projection of the DMH to the parabrachial nucleus was searched for, it was found upon close examination either nonexistent or weak [60]–[62]. Likewise, earlier reports of a direct projection of the DMH to the insular cortex [55] were not confirmed in subsequent studies using retrograde labeling [61], [63].

Whereas DMH-lesioned and PVH-lesioned animals seem fully capable of both sensing thermal information and responding to it with an appropriate locomotor act, they fail to exhibit cold-seeking behavior when treated with a high dose of LPS. It is, therefore, likely that the DMH and PVH are involved in a stimulus-specific, affective or motivational (but not sensory or motor), component of LPS-induced cold-seeking behavior. In support, the DMH sends marked projections to the septum and amygdala [55], [61], [64], and these telencephalic structures are well documented to mediate the affective or motivational component of various behaviors [65]–[67]. Furthermore, the septum drives warmth-induced locomotion in rats, as found by Roberts and Mooney [68] in their elegant experiments involving localized diathermic warming of five distinct brain areas. Importantly, the proposed involvement of the DMH projections to the septum and amygdala explains not only the effect of DMH ablation on LPS-induced cold-seeking behavior, but also the effect of PVH ablation. One of the major routes for ascending projections from the DMH is through the hypothalamic periventricular zone [61], [69], and this route is likely to be interrupted by electrocoagulation (but not lesioning with ibotenic acid) of the PVH. Hence, we propose that DMH neurons sending their axons to the telencephalon (perhaps the septum and amygdala) through the PVH participate in the affective or motivational (but not in the sensory or motor) component of LPS-induced cold-seeking behavior.

In conclusion, his study identifies two neural elements essential for the cold-seeking response of rats to bacterial LPS: neuronal bodies located in the DMH and axons passing through the PVH. These are the first two landmarks on the map of the circuitry of cold-seeking behavior associated with endotoxin shock.

## Methods

### Animals

Experiments were performed in adult male Wistar rats (Harlan, Indianapolis, IN, USA). At the time of the electrolytic or excitotoxic lesioning, the rats had a body mass of 370–400 g. The rats were housed in individual cages in a rack equipped with a Smart Bio-Pack ventilation system and Thermo-Pak temperature control system (Allentown Caging Equipment, Allentown, NJ, USA); the incoming air temperature was maintained at 28°C. The room was on a 12:12 h light-dark cycle (lights on at 7:00 A.M.). The animals had free access to tap water and standard rat chow (Harlan Teklad, Madison, WI, USA). Before the experiments, the rats were habituated extensively (4 daily training sessions, 6–16 h each) to stay in a thermogradient apparatus (described below). On the day before the experiment, the rats were placed in the apparatus at 6:00 PM and further acclimated to the experimental conditions by staying in the apparatus overnight. All injections and other experimental interventions were made the next day, between 8:00 AM and 3:00 PM. The experimental protocols were approved by the St. Joseph's Hospital Animal Care and Use Committee.

### Electrolytic lesions

All surgical and lesioning procedures were performed under ketamine-xylazine-acepromazine (55.6, 5.5, and 1.1 mg/kg, respectively, intraperitoneally) anesthesia and antibiotic (enrofloxacin, 1.2 mg/kg, subcutaneously) protection. All brain lesions were placed on *Day 0*. For electrolytic lesioning, a rat was anesthetized, and the skin of the head over the frontal and parietal bones was shaved and scrubbed. The rat was fixed to a stereotaxic apparatus (David Kopf, Tujunga, CA, USA) with the incisor bar set 3.3 mm ventral to interaural line. The skin was incised over the sagittal suture, the skin and the underlying muscles were retracted, and the periosteum was detached from the bone and excised. A stainless steel electrode (250 µm diameter, Frederic Haer, Bowdoinham, ME, USA) was inserted into the brain. All stereotaxic coordinates were taken from Paxinos and Watson [70], but the anteroventral coordinate was increased by 0.5 mm to adjust for the difference in the body mass between the rats used by Paxinos and Watson [70] and those used in the present study. The following stereotaxic coordinates were used: 0.0 mm (at bregma), 0.5 mm from the midline, and 8.5 mm from the skull surface for the POA; −1.5 mm from bregma, 0.5 mm from the midline, and 8.7 mm from the skull surface for the PVH; and −2.8 mm from bregma, 0.5 mm from the midline, and 9.0 mm from the skull surface for the DMH. A second electrode (an “alligator” clip) was attached to the edge of the surgical wound on the head. To lesion the brain tissue, a precision lesioning instrument (Ugo Basile, Comerio, Italy) was used. A constant anodal current (1 mA) was passed through the electrodes for 10 s (PVH) or 30 s (POA or DMH). After the structure of interest was lesioned on one side, the electrode was removed and inserted at the same coordinates contralaterally. Sham-lesioned rats were prepared similarly, but the tip of the electrode was placed 2 mm above the POA, PVH, or DMH, and no current was passed.

### Chemical lesions

Unless specified otherwise, all drugs and reagents were purchased from Sigma-Aldrich (St Louis, MO, USA). To prevent the hypertensive and other cardiovascular side effects of intrabrain administration of ibotenic acid, the rats were pretreated with the ganglionic blocker hexamethonium (30 mg/kg, intraperitoneally). For lesioning the PVH or DMH, a glass micropipette (tip diameter, 50 µm) was inserted into the target area by using the same coordinates as for electrocoagulation. A solution of ibotenic acid (10 µg/µl) in phosphate-buffered (0.01 M, pH 7.4) saline was infused with the help of an infusion pump (30 nl/min) over 3.3 min (to deliver the total volume of 100 nl; PVH) or 10 min (300 nl; DMH), and the pipette was left in place for 5 min. Sham-lesioned animals were infused with vehicle. All infusions were performed bilaterally.

All rats with electrolytic or excitotoxic brain lesions were examined daily for signs of dehydration and general malaise. If a rat had lost more than 15% of its body mass 24 h after lesioning or was failing to recover from its initial loss of body mass, it was injected with isotonic saline (10 ml, subcutaneously) to counteract dehydration.

### Jugular catheterization and temperature datalogger implantation

On *Day 10*, each animal was anesthetized a second time. After a midline laparotomy was performed, a miniature temperature datalogger (SubCue, Calgary, Alberta, Canada) was inserted in the peritoneal cavity and sutured to the lateral abdominal wall. The datalogger had been programmed to acquire data (measure T_b_) every 5 min. The surgical wound was sutured in layers. The rat was then implanted with a jugular vein catheter. A 1-cm longitudinal incision was made on the ventral surface of the neck, 1 cm left of the trachea. The left jugular vein was exposed, freed from its surrounding connective tissue, and ligated. A silicone catheter (ID 0.5 mm, OD 0.9 mm) filled with heparinized (10 U/ml) pyrogen-free saline was passed into the superior vena cava through the jugular vein and secured in place with ligatures. The free end of the catheter was knotted, tunneled under the skin to the nape, and exteriorized. The surgical wound on the ventral surface of the neck was sutured. The jugular catheter was flushed with heparinized saline (10 U/ml) the day after surgery and every other day thereafter. The experiments were performed on *Days 13*–*19*.

### Thermogradient apparatus

The six-channel thermogradient apparatus used is described in detail elsewhere [5]. In brief, the apparatus was built from 12-mm-thick aluminum sheets welded together to form six parallel channels running between two tanks. Each channel (200 long x 12 wide x 20 cm high) had an adjustable stainless steel grid (that served as a floor for the animals tested) and was covered with an acrylic double-panel lid. The tank at one end of the apparatus was filled with water and equipped with two heating units (PolyScience, Niles, IL, USA); the tank at the other end was constantly perfused with 10% ethylene glycol by an external-circulation cooling/heating pump (PolyScience). This setting allowed an almost linear longitudinal T_a_ gradient common for all channels to be established. In the present study, T_a_ ranged from 15°C at the cold end to 30°C at the warm end; T_a_ changed with a change in the longitudinal position in the channel at a rate of 0.15°C/cm. Within each channel, T_a_ was monitored by five evenly spaced (50 cm apart) thermocouples located under the grid floor, and the position of the rat was monitored by 56 evenly spaced (3.5 cm) infrared emitter-receiver pairs, which formed transversal infrared beams.

### Studying behavioral thermoregulation

As reported elsewhere [5], thermoregulatory behavior was induced by inflammatory, thermal, or pharmacological stimuli. Inflammatory stimulation was achieved by injecting LPS (*Escherichia coli*, serotype 0111:B4) through the jugular catheter at a low dose (10 µg/kg; to cause warmth-seeking behavior) or a high dose (5,000 µg/kg; to cause cold-seeking behavior). Thermal stimulation was achieved by confining each rat to a short (22 cm) portion of the channel near either the warm end (T_a_ of ∼28°C in the middle of the confinement zone) or the cold end (T_a_ of ∼17°C in the middle of the confinement zone) of the thermogradient apparatus for 1 or 2 h. Both the preferred T_a_ and the midpoint of the thermoneutral zone for rats in this apparatus are ∼24°C [5]. Therefore, confining a rat at 28 or 17°C results in mild heat exposure or moderate cold exposure, respectively. Pharmacological induction of cold-seeking behavior was attempted by injecting the rats with RTX (0.5 µg/kg, i.v.), a TRPV1 agonist. Pharmacological induction of warmth-seeking behavior was attempted by injecting the rats with menthol (50 µg/kg, i.v.), a TRPM8 agonist. Both RTX and menthol were dissolved in saline containing 10% ethanol and 10% propylene glycol.

### Testing autonomic defense of body temperature

The ability of rats to defend their T_b_ (recorded by the dataloggers implanted in the peritoneal cavity) during heat or cold exposure was tested. These tests were performed the same way as thermal stimulation for behavioral experiments (see above). In brief, each rat was confined to a short portion of the channel near the warm (∼28°C) or cold end (∼17°C) of the thermogradient apparatus. The rats could not move to a different T_a_; therefore, they were forced to regulate their T_b_ mostly by autonomic mechanisms.

### Histological verification

To verify the correct placement of the lesions, the rats were anesthetized and perfused through the ascending aorta (right atrium cut) with saline (50 ml, 5 min) followed by 10% formalin (50 ml, 5 min). The brains were removed, placed in phosphate-buffered (0.1 M, pH 7.4) saline containing 30% sucrose and 10% formalin, and post-fixed in this solution at 4°C for 48 h. The brains were then frozen in dry ice and sectioned (50 µm). Sections containing the structures of interest were collected, mounted on glass slides, stained either with cresyl-violet (stains the Nissl bodies in the cytoplasm of neurons purple-blue) or Klüver-Barrera stain (stains the myelinated fibers blue and the cell bodies violet), and examined under a light microscope.

### Data processing and analysis

The preferred T_a_ was calculated based on a linear relationship between the position in the channel and T_a_. A weighted average was calculated for every 5-min interval. The obtained curves of preferred T_a_ were smoothed by a second polynomial degree Savitzky-Golay filtering over a 30-min shifting window using Microcal Origin 5.0 software (OriginLab, Northampton, MA, USA). The T_b_ and preferred T_a_ responses were compared across treatments and time points by a two-way analysis of variance for repeated measurements followed by the Tukey test (Sigma Stat, Systat Software, Point Richmond, CA, USA). The differences were considered significant at P<0.05. The data are reported as mean±standard error.
